# Overexpression of *GmNFYA5* confers drought tolerance to transgenic Arabidopsis and soybean plants

**DOI:** 10.1186/s12870-020-02337-z

**Published:** 2020-03-20

**Authors:** Xiao-Jun Ma, Tai-Fei Yu, Xiao-Hui Li, Xin-You Cao, Jian Ma, Jun Chen, Yong-Bin Zhou, Ming Chen, You-Zhi Ma, Jun-Hua Zhang, Zhao-Shi Xu

**Affiliations:** 1 College of Agronomy, Northeast Agricultural University, Harbin, 150030 China; 2 Institute of Crop Science, Chinese Academy of Agricultural Sciences (CAAS)/National Key Facility for Crop Gene Resources and Genetic Improvement, Key Laboratory of Biology and Genetic Improvement of Triticeae Crops, Ministry of Agriculture, Beijing, 100081 China; 3 Crop Germplasm Resources Institute, Jilin Academy of Agricultural Sciences, Gongzhuling, 136100 China; 4 Crop Research Institute, Shandong Academy of Agricultural Sciences, National Engineering Laboratory for Wheat and Maize, Key Laboratory of Wheat Biology and Genetic Improvement, Jinan, 250100 China; 5 College of Agronomy, Jilin Agricultural University, Changchun, 130118 China

**Keywords:** ABA sensitivity, *Glycine max*, Nuclear factor YA, Resistance mechanisms

## Abstract

**Background:**

Crop productivity is challenged by abiotic stresses, among which drought stress is the most common. *NF-Y* genes, especially *NF-YA* genes, regulate tolerance to abiotic stress.

**Results:**

Soybean NF-Y gene *GmNFYA5* was identified to have the highest transcript level among all 21 *NF-YA* genes in soybean (*Glycine max* L.) under drought stress. Drought-induced transcript of *GmNFYA5* was suppressed by the ABA synthesis inhibitor naproxen (NAP). *GmNFYA5* transcript was detected in various tissues at vegetative and reproductive growth stages with higher levels in roots and leaves than in other tissues, which was consist with the *GmNFYA5* promoter: GUS fusion assay. Overexpression of *GmNFYA5* in transgenic Arabidopsis plants caused enhanced drought tolerance in seedlings by decreasing stomatal aperture and water loss from leaves. Overexpression and suppression of *GmNFYA5* in soybean resulted in increased and decreased drought tolerance, respectively, relative to plants with an empty vector (EV). Transcript levels of ABA-dependent genes (*ABI2*, *ABI3*, *NCED3*, *LEA3*, *RD29A*, *P5CS1*, *GmWRKY46*, *GmNCED2* and *GmbZIP1*) and ABA-independent genes (*DREB1A*, *DREB2A*, *DREB2B*, *GmDREB1*, *GmDREB2* and *GmDREB3*) in transgenic plants overexpressing *GmNFYA5* were higher than those of wild-type plants under drought stress; suppression of *GmNFYA5* transcript produced opposite results. GmNFYA5 probably regulated the transcript abundance of *GmDREB2* and *GmbZIP1* by binding to the promoters in vivo.

**Conclusions:**

Our results suggested that overexpression of *GmNFYA5* improved drought tolerance in soybean via both ABA-dependent and ABA-independent pathways.

## Background

Crop plants are commonly affected by abiotic stresses which cause soil destruction and crop losses worldwide [1, 2]. Water stress, either caused by drought or salt, is a major challenge. Drought is more widespread and damaging than salt stress [3]. Adaptation to drought involves complex regulatory networks involving control of water flux and cellular osmotic adjustment [4–6]. ABA regulates drought tolerance in plants by regulating stomatal aperture and modulating drought-responsive genes such as AP2/ERF and MYB family members that play important roles under drought stress [7–9]. ABA synthesis inhibitors such as naproxen and tungstate show the importance of ABA [10, 11].

The CCAAT box binding factor (CBF), also known as Nuclear Factor Y (NF-Y) or Heme Activator Protein (HAP), is composed of subunits NF-YA, NF-YB and NF-YC [12–14]. In monocotyledonous and dicotyledonous plants, just one of these subunits is encoded by tens of genes, whereas only one or two genes are present in animals [13]. Members of the different NF-Y families have diverse functions in regulating plant development and growth, such as flowering time, plant height, root elongation, embryogenesis, and seed germination [15–26].

The NF-Y genes are regulators of abiotic stress-induced responses. For example, overexpression of *AtNFYA5* in Arabidopsis caused enhanced resistance to drought stress by affecting the expression of many drought stress-related genes [27]. Transgenic rice lines carrying extra copies of *OsNFYA7* showed improved drought tolerance through the ABA-independent pathway [28]. Transgenic rice overexpressing *OsHAP2E* [29] and bermudagrass *Cdt-NFYC1* [11] displayed improved resistance to drought and salt stresses. Several stress-related measures showed that overexpression of *ZmNFYB2* resulted in drought resistance in transgenic maize plants [30]. However, most reports showing that NF-Ys play important roles under water stress conditions relate to rice and Arabidopsis. The functions of few NF-Y members were confirmed in soybean. *GmNFYA3* and *GmNFYB1* were induced by drought and transgenic Arabidopsis lines with those genes showed increased tolerance to drought stress [31, 32]. However, the biological functions of many other NF-Y members in soybean remain to be verified.

In this study, *GmNFYA5*, a soybean member of the NF-YA family, was induced by drought and ABA. Transgenic Arabidopsis and soybean lines overexpressing *GmNFYA5* showed enhanced tolerance to drought stress through both ABA-dependent and ABA-independent pathways. Considering these results, we suggest that *GmNFYA5* is an excellent candidate gene for genetic improvement to enhance drought tolerance of soybean.

## Results

### Isolation and characterization of *GmNFYA5*

**Fig. 1:**
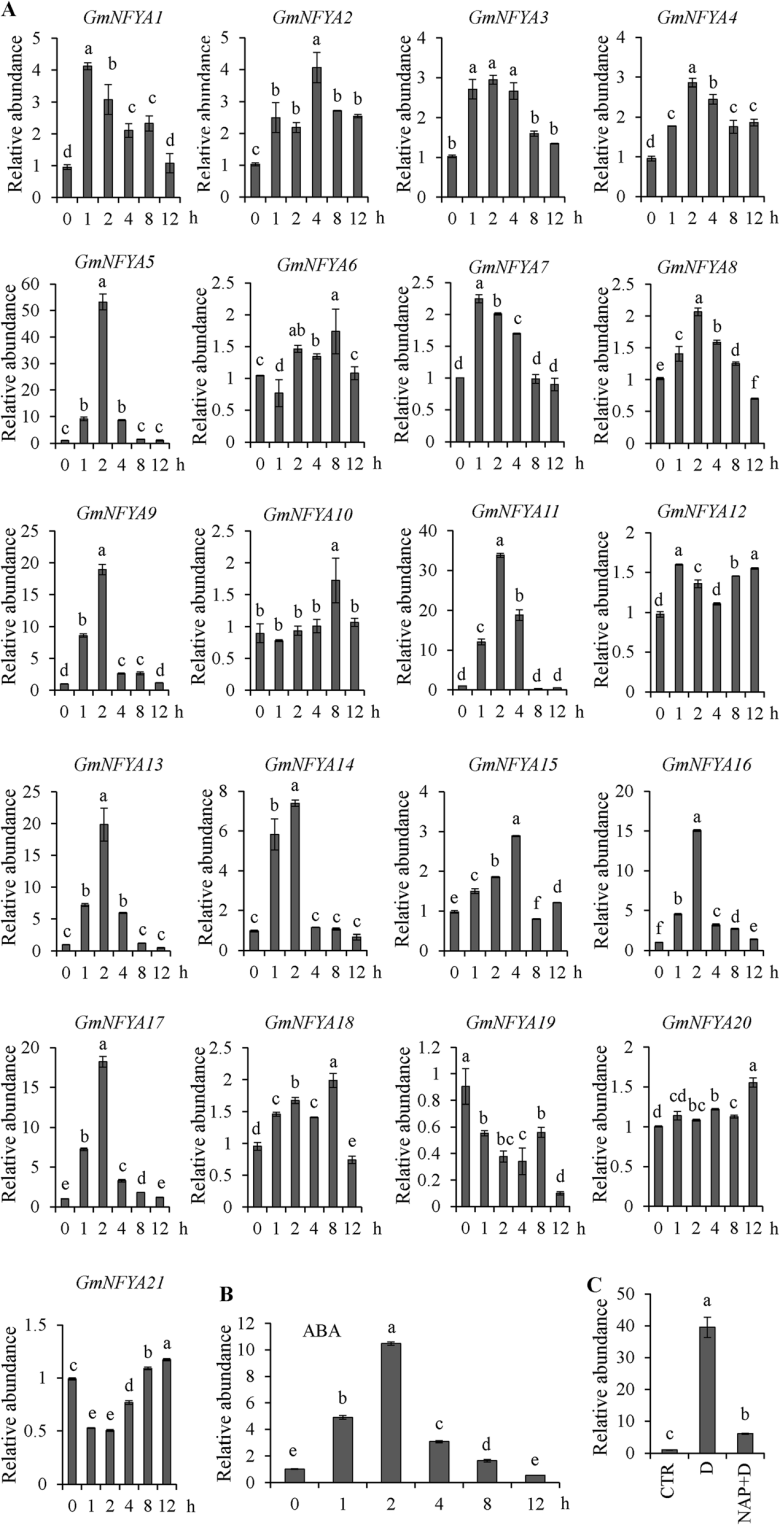
*GmNFYA5* expression as affected by drought stress and ABA. (A) Transcripts of 21 NF-YA genes were analyzed using qRT-PCR in soybean under drought stress. Expression levels were normalized to that of *GmCYP2*. *GmNFYA5* transcript at 0 h was set at 1.0, and SD for three biological replicates is represented by error bars. (B) Transcript of *GmNFYA5* analyzed in response to ABA treatment in soybean. Expression levels were normalized to that of *GmCYP2*. *GmNFYA5* transcript at 0 h was set at 1.0, and SD for three biological replicates is represented by error bars. (C) qRT-PCR of *GmNFYA5* transcript in soybean plants in response to drought stress with the treatment of 1 mM NAP. Control, drought and drought + NAP are indicated by CTR, D and D + NAP, respectively. The level of *GmNFYA5* transcript under control conditions was set at 1.0, and the internal control was *GmCYP2*. SD for three biological replicates is represented by error bars. Significant differences at *P* < 0.05 are indicated by different letters above the columns

*NF-YA* genes mediate in drought stress tolerance [27, 28, 31]. Twenty one *NF-YA* genes were investigated in seedling leaves using qRT-PCR and most of them were induced by drought stress. *GmNFYA5* transcript was highest among all genes at 2 h after drought stress (Fig. 1A) and transcription of *GmNFYA5* was significantly induced by ABA (Fig. 1B). Detached leaves were used to understand whether ABA could affect the drought-induced transcript of *GmNFYA5* by using NAP, an inhibitor of ABA biosynthesis. The *GmNFYA5* transcript under drought treatment for 2 h was similar to the above pattern relative to the control (Fig. 1A and C). However, pretreatment with NAP suppressed the level of expression of *GmNFYA5* (Fig. 1C).

*GmNFYA5* encodes a 37.68 KD polypeptide of 303 amino acid residues with an isoelectric point (p*I*) of 8.86. GmNFYA5 contains highly conserved core regions in common with Arabidopsis NF-YA proteins; these consist of two subdomains: an NF-YB/C and DNA binding subdomains connected by a linker and required for association with the NF-YB/C heterodimer, and CCAAT binding sequences. The amino acids indicated by asterisks in Fig. S1A are critical, conserved residues. Alignments of GmNFYA5 and Arabidopsis NF-YA proteins showed that it had the highest identity with AtNFYA5 in the conserved domain. Phylogenetic analysis based on the amino acid sequences in the conserved domain showed that GmNFYA5 clustered with Arabidopsis AtNFYA6 and AtNFYA5 (Fig. S1B).

### Tissue-specific expression analysis and subcellular localization

**Fig. 2:**
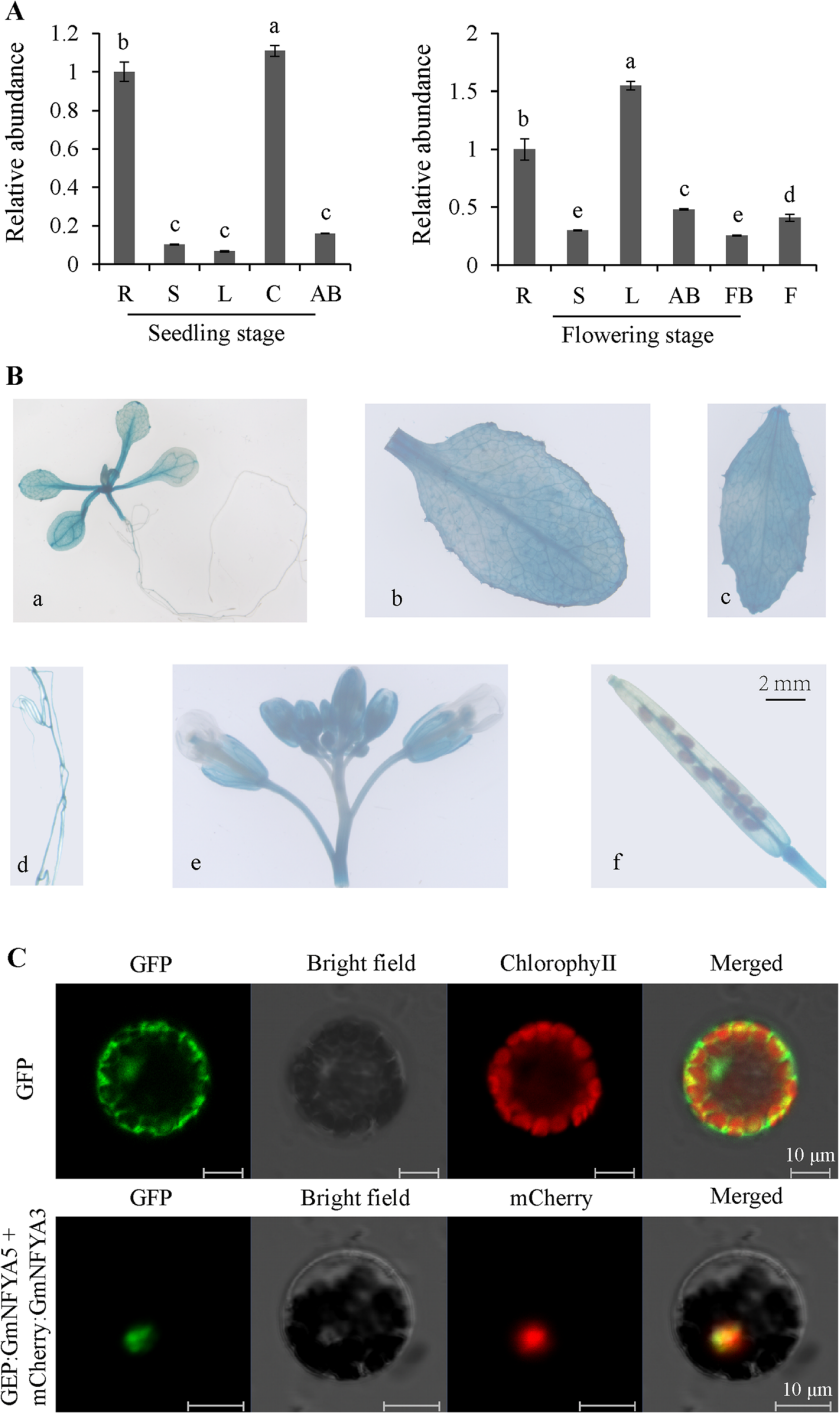
Tissue specific expression analysis and subcellular localization of *GmNFYA5*. (A) Expression abundance of the *GmNFYA5* gene in soybean tissues at the seedling and flowering stages. Soybean tissues under normal conditions were used to extract total RNA. The relative transcript levels of *GmNFYA5* in soybean tissues are indicated by *vertical* columns. *GmNFYA5* transcript in roots was set at 1.0, and the internal control was *GmCYP2.* SD for three biological replicates is represented by error bars. Significant differences at *P* < 0.05 are indicated by different letters above the columns. (B) Expression pattern of *GmNFYA5* promoter: GUS in various tissues of transgenic Arabidopsis plants. *a* 5-day-old seedling, *b* rosette leaf, *c* cauline leaf, *d* root, *e* flower, *f* silique. Scale bar, 2 mm (C) Subcellular localization of GmNFYA5. Fluorescence of GFP-GmNFYA5 and mCherry-GmNFYA3 fusion proteins in transformed cells was localized exclusively to the nucleus. Scale bar, 10 μm

The expression of *GmNFYA5* in different tissues, including roots, stems, cotyledon, leaves, apical buds, flower buds and flowers was evaluated by qRT-PCR at vegetative and reproductive growth stage under normal conditions. *GmNFYA5* transcripts were detected in every tissue at both developmental stages, with a significant increase in leaves at flowering compared with seedling levels (Fig. 2A). Transcript abundance in roots and leaves was higher than in other tissues (Fig. 2A). To investigate the expression patterns in greater detail, transgenic Arabidopsis T_3_ lines overexpressing *GmNFYA5* promoter: GUS were produced. Expression of GUS was detected in various tissues (Fig. 2B). *GmNFYA5* expression was highest in the floral tissues, but also prominent in the leaf vascular system and roots. As to leaves, the lower level of GUS staining in seedlings and higher level of staining at the flowering stage (Fig. 2B) were consistent with the results of the qRT-PCR.

To investigate the localization of GmNFYA5 within cells, recombinant vectors p16318GFP:*GmNFYA5* and mCherry:*GmNFYA3* were co-transformed into Arabidopsis protoplasts. GFP and mCherry fluorescence of the two fusion protein in transformed cells were localized exclusively to the nuclei (Fig. 2D), whereas the control GFP was uniformly distributed across protoplast cells (Fig. 2C), thus showing that GmNFYA5 was a nuclear-localized protein.

### Sensitivity of *35S:GmNFYA5* Arabidopsis plants to exogenous ABA and PEG

**Fig. 3:**
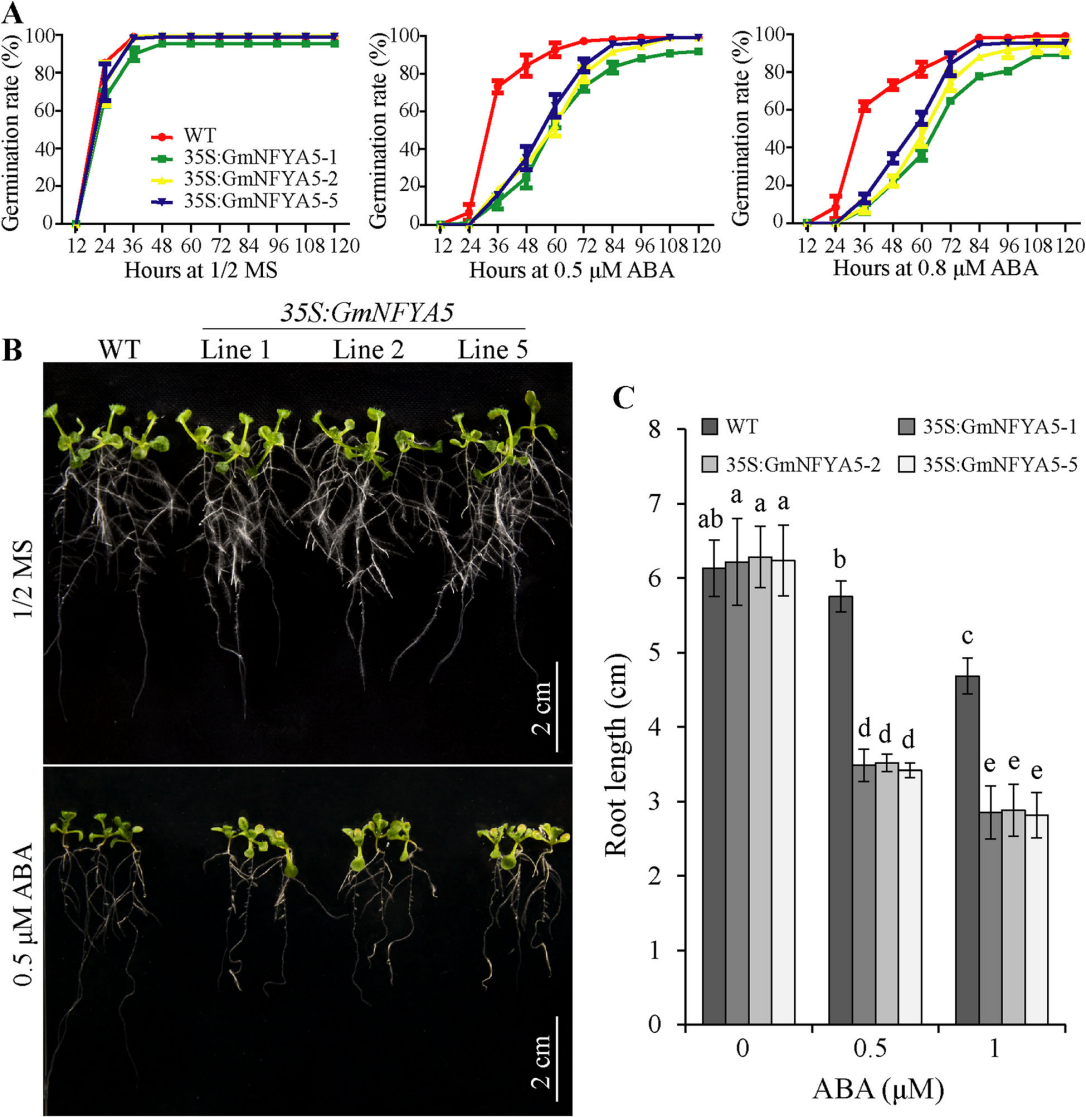
Germination and root growth of WT and *35S:GmNFYA5* Arabidopsis plants with ABA treatment. (A) Seed germination on 1/2 MS agar plates with 0, 0.5 and 0.8 μM ABA. Each measurement represents the average germination of 64 seeds ± SD. (B) and (C) Root growth of WT and *35S:GmNFYA5* Arabidopsis plants on media with/without ABA. Three-day-old seedlings from 1/2 MS medium were transferred to media containing 0, 0.5 and 1 μM ABA; and photographed 7 days later. Scale bar, 2 cm. Each measurement represents the average root length of 30 seedlings ± SD. Significant differences P < 0.05 are indicated by different letters above the columns

Transgenic Arabidopsis plants overexpressing *GmNFYA5* were generated to elucidate functions of the gene. Three homozygous transgenic lines (*35S:GmNFYA5–1*, *35S:GmNFYA5–2* and *35S:GmNFYA5–5*) detected using qRT-PCR were selected for functional analysis (Fig. 5B). To determine whether ABA sensitivity was affected by *GmNFYA5*, seeds from *35S:GmNFYA5* and WT Arabidopsis plants were germinated on 1/2 MS medium with different concentrations of ABA. Germination rates were similar under control conditions (Fig. 3A). In the presence of 0.5 and 0.8 μM ABA seed germination was significantly suppressed in both *35S:GmNFYA5* and WT lines, but the inhibition of WT germination was much less than *35S:GmNFYA5* lines; germination of WT seeds reached 73% at 36 h compared with 12 to 19% for *35S:GmNFYA5* lines treated with 0.5 μM ABA (Fig. 3A). Only 7 to 13% germination was detected in *35S:GmNFYA5* lines compared to 62% for WT seeds at 36 h in treatments with 0.8 μM ABA (Fig. 3A).

Differential inhibition of root growth between WT and *35S:GmNFYA5* seedlings was also observed following treatment with ABA. Three-day-old seedlings under normal conditions were transferred to1/2 MS medium with 0.5–1 μM ABA. After one week, roots lengths of *35S:GmNFYA5* plants were shorter than WT plants (Fig. 3B and C).

**Fig. 4:**
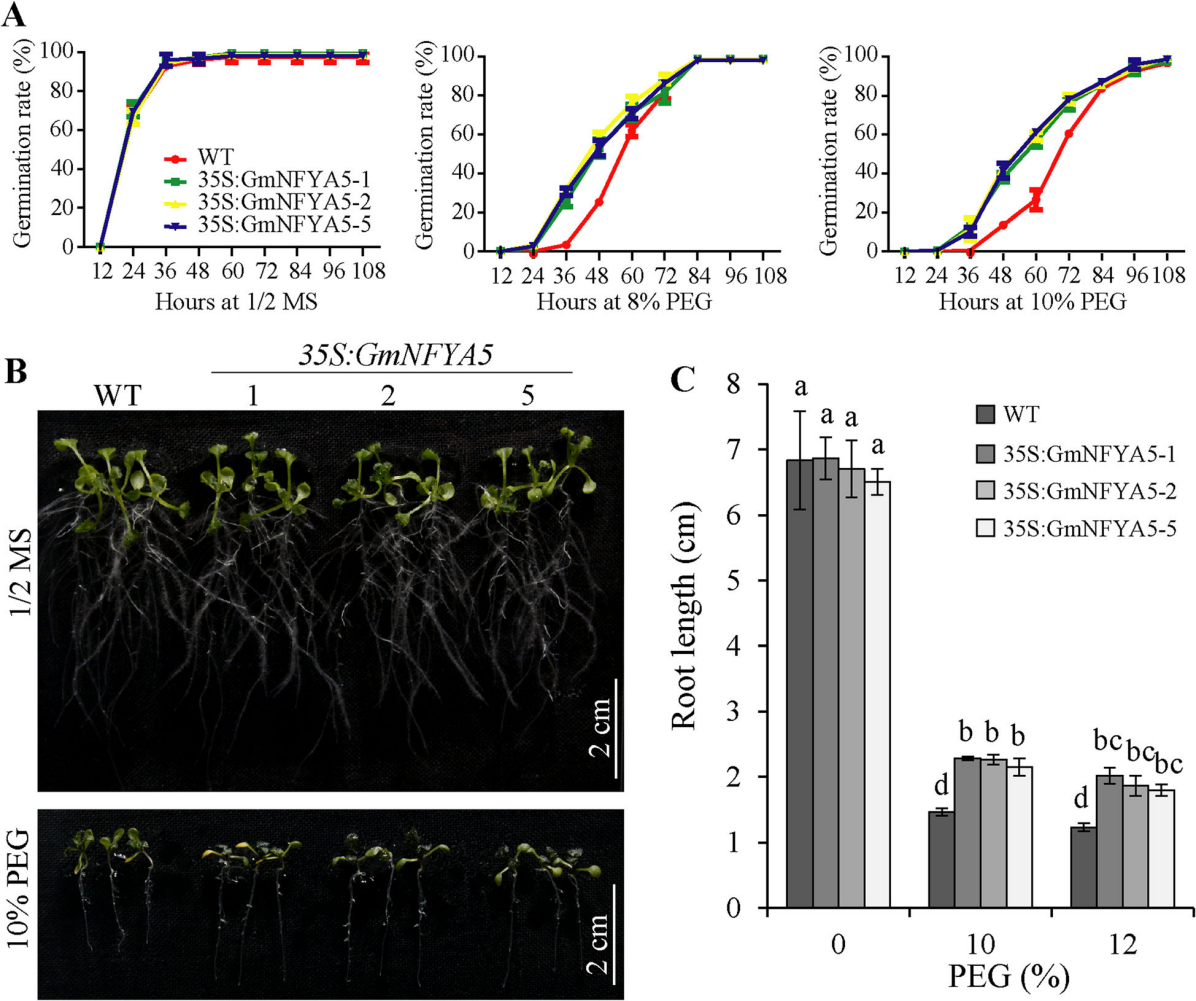
Germination rates and root growth of WT and *35S:GmNFYA5* Arabidopsis plants treated with PEG. (A) Seed germination on 1/2 MS agar plates with 8 and 10% PEG. Each measurement represents the average germination of 64 seeds ± SD. (B) and (C) Root growth of WT and *35S:GmNFYA5* Arabidopsi*s* plants on media with/without PEG. Three-day-old seedlings from 1/2 MS medium were transferred to media containing 0, 10 and 12% PEG; photographed after 7 days. Scale bar, 2 cm. Each measurement represents the average root length of 30 seedlings ± SD. Significant differences at P < 0.05 are indicated by different letters above the columns

The effects of different concentrations of PEG on germination of WT and *35S:GmNFYA5* lines were also assessed. Germination of WT seeds was more sensitive to PEG than *35S:GmNFYA5* lines. Compared to 53–59% germination of *35S:GmNFYA5* Arabidopsis seeds at 48 h in 8% PEG, there was only 25% germination of WT seeds (Fig. 4A). Germination rates of WT seeds were further delayed relative to *35S:GmNFYA5* Arabidopsis seeds in 10% PEG (Fig. 4A). Seedling growth was also differentially inhibited by PEG (Fig. 4B and C).

### Tolerance of *35S:GmNFYA5* Arabidopsis seedlings to drought stress

**Fig. 5:**
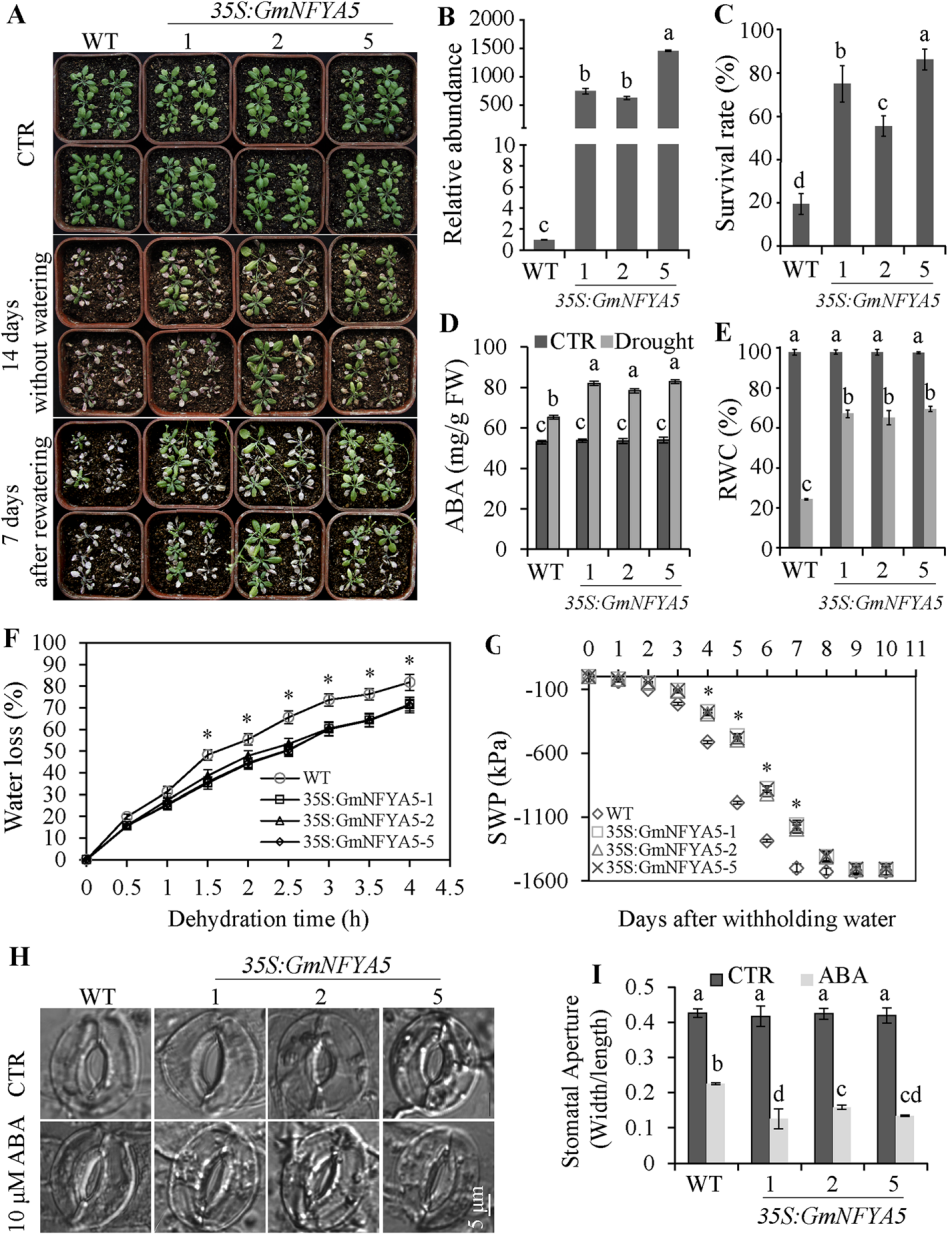
Improved drought tolerance and stomatal aperture analysis in *35S:GmNFYA5* Arabidopsis plants. (A) Assessment of drought tolerance in *35S:GmNFYA5* Arabidopsis plants. Three-week-old plants were grown without water for 14 days, followed by re-watering for 7 days. Drought resistance of *35S:GmNFYA5* Arabidopsi*s* plants was assayed by capability to resume growth when returned to normal conditions after drought stress. (B) *GmNFYA5* transcript detected in *35S:GmNFYA5* Arabidopsis lines. Expression levels were normalized to that of *Tub8*. Transcript of *GmNFYA5* in WT plants was set at 1.0; SD for three biological replicates is represented by error bars. (C-G) Measurements of survival rate, concentrations of ABA, RWC, water loss, and SWP in *35S:GmNFYA5* Arabidopsis and WT lines. (H-I) Stomatal apertures in *35S:GmNFYA5* Arabidopsis and WT plants with zero and 10 μM ABA treatments. Width/length of the stomatal aperture was measured using the ruler tool in Adobe Photoshop CS5. Scale bar, 5 μm. Data represent mean SD for three biological replicates (*n* = 54). Significant differences P < 0.05 are indicated by different letters above the columns

To determine whether overexpression of *GmNFYA5* enhanced drought tolerance, 3-week-old *35S:GmNFYA5* and WT Arabidopsis seedlings were deprived of water. The soil water potential (SWP) of WT seedlings fell more quickly than *35S:GmNFYA5* lines (Fig. 5G). The SWP of WT and *35S:GmNFYA5* lines declined to their minimum levels after drought stress for 7 and 9 days, respectively. Detached leaves of *35S:GmNFYA5* plants also lost water more slowly than the WT (Fig. 5D). Concentrations of ABA in the *35S:GmNFYA5* lines were higher than in WT plants after drought treatment for 7 days, and there was no significant difference when the plants were grown under normal conditions (Fig. 5D). Consistent with these results, the stomatal apertures of detached leaves from *35S:GmNFYA5* lines were significantly smaller than those from the WT under the treatment with 10 μM ABA compared with no significant difference in non-treated leaves (Fig. 5h and i).

Drought treatment for 14 days produced more extreme wilting in WT plants than the *35S:GmNFYA5* lines (Fig. 5A). The *35S:GmNFYA5* plants maintained 65–69% RWC and 54–62% ion leakage compared to 24% RWC and 79% ion leakage in leaves of WT plants. No significant difference was detected among *35S:GmNFYA5* and WT controls under normal conditions (Fig. 5E and Fig. S3). After re-watering for 7 days, most WT plants failed to recover (19% survival), whereas most *35S:GmNFYA5* plants maintained turgor and showed higher recovery (56–86% survival) (Fig. 5C). These results showed that overexpression of *GmNFYA5* enhanced drought tolerance of transgenic Arabidopsis lines.

### Drought tolerance of *35S:GmNFYA5* Arabidopsis lines is maintained for the entire growth cycle

**Fig. 6:**
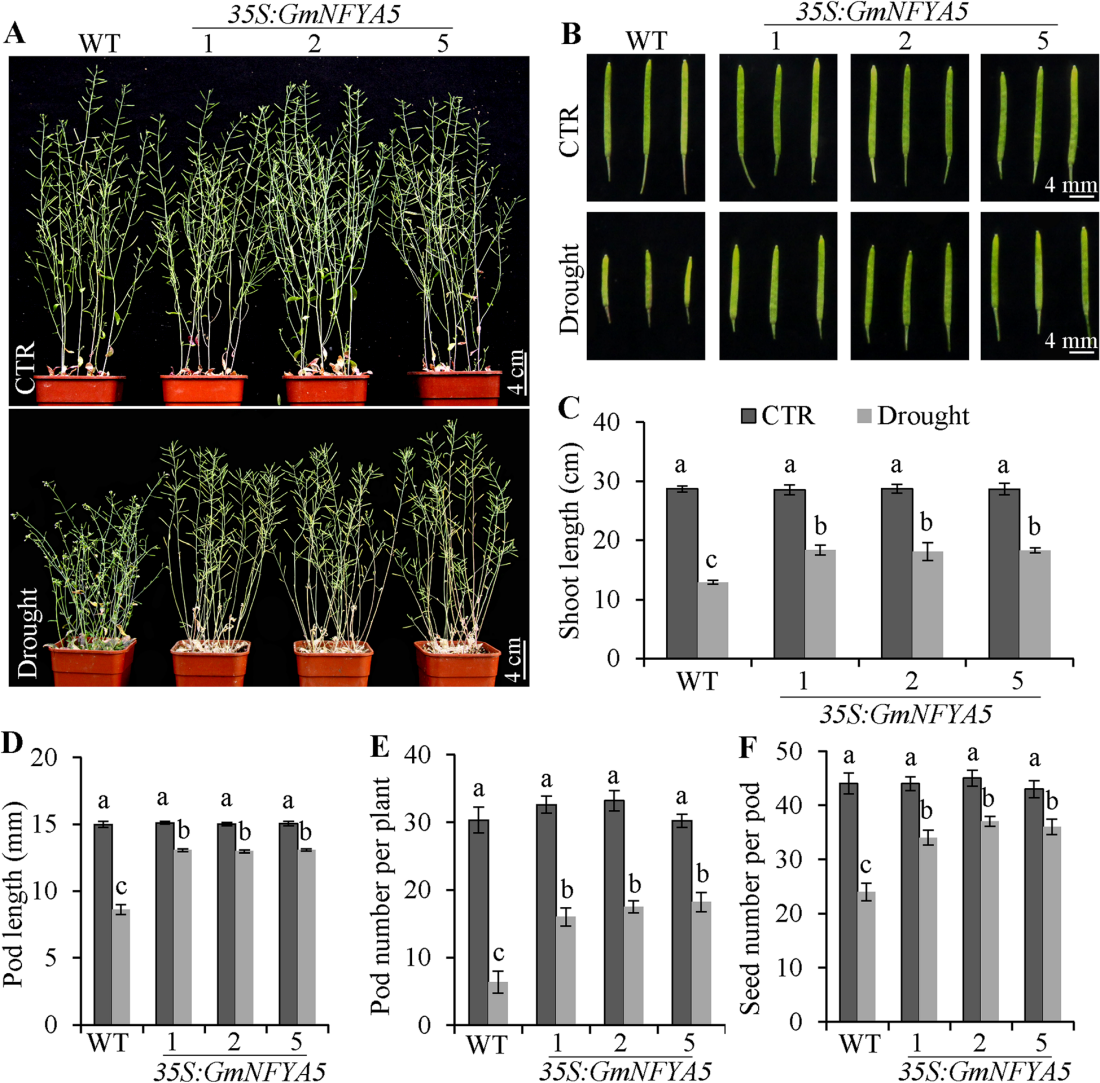
Improved drought tolerance in *35S:GmNFYA5* Arabidopsis plants all over an entire growth cycle. (A) Drought tolerance assessed in *35S:GmNFYA5* Arabidopsis over a full growth cycle. Scale bar, 4 cm. (B) Pod lengths in *35S:GmNFYA5* and WT Arabidopsis plants were from control and drought conditions. Scale bar, 4 mm. (C-F) Shoot length, pod length, pod number per plant and seed number per pod were measured in *35S:GmNFYA5* and WT plants under control and drought conditions. Data represent mean SD for three biological replicates (*n* = 18). Significant differences at P < 0.05 are indicated by different letters above the columns

*GmNFYA5* overexpression increased drought tolerance of *35S:GmNFYA5* Arabidopsis lines at the seedling stage. However, this increase had to be translated to enhanced drought tolerance all over an entire growth cycle and ultimately lead to increased yield of dry matter or seed. Under continued drought treatment the shoot and pot lengths of *35S:GmNFYA5* plants were markedly longer (Fig. 6A-D), and pod numbers per plant and seed numbers per pod were significantly higher than the WT (Fig. 6E and F). There was no difference between *35S:GmNFYA5* and WT plants grown under normal conditions. These results showed that *GmNFYA5* overexpression in Arabidopsis plants could improve drought tolerance and seed yields relative to the WT control.

### Transcription profiles of stress-related genes in *35S:GmNFYA5* Arabidopsis

**Fig. 7:**
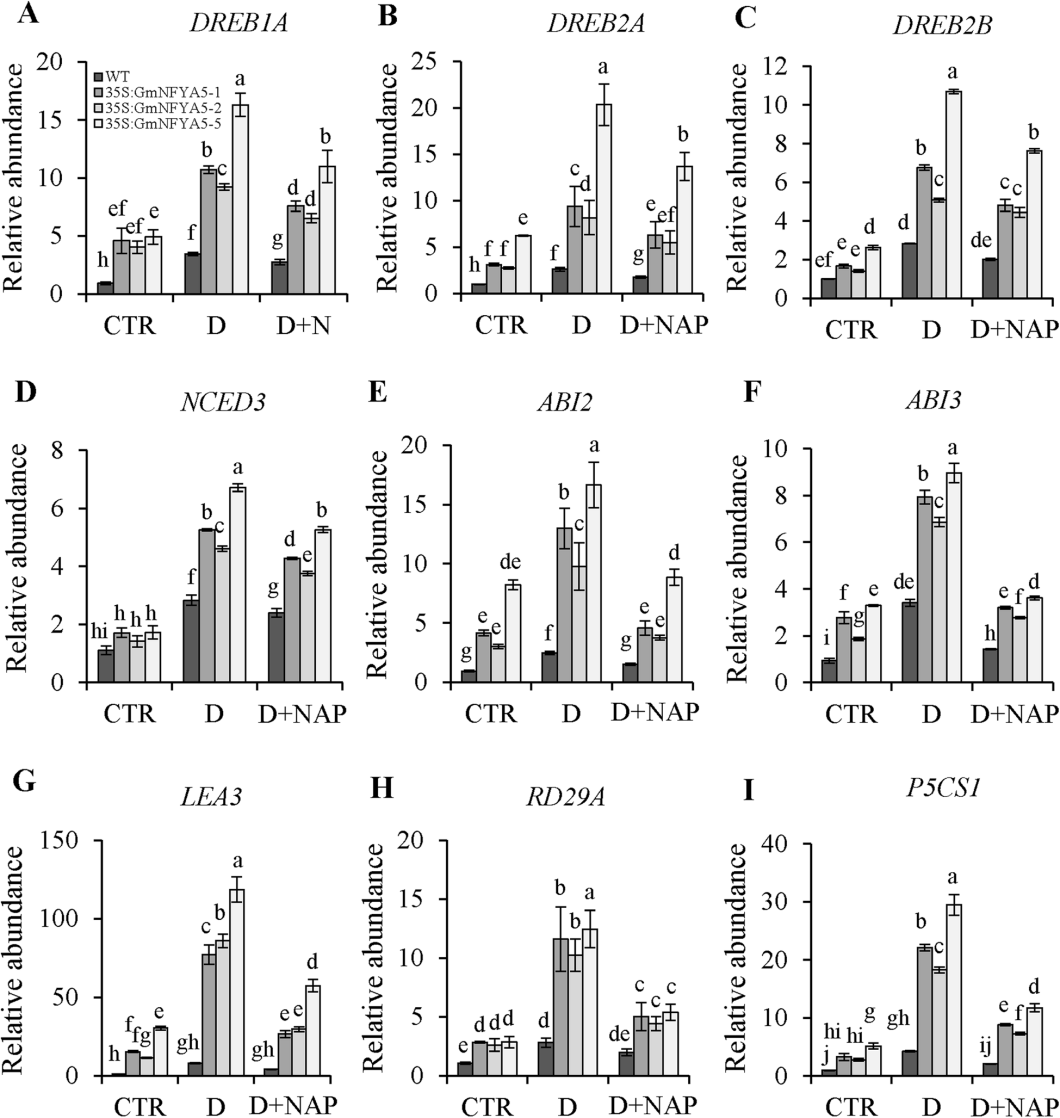
Relative transcript levels of *DREB1A*, *DREB2A*, *DREB2B*, *ABI2*, *ABI3*, *NCED3*, *LEA3*, *RD29A* and *P5CS1* in *35S:GmNFYA5* and WT Arabidopsis plants under three conditions. Relative transcript level is indicated by the *vertical* column and normalized to that of *Tub8*. Transcripts of stress-related genes in WT plants under normal conditions were set at 1.0. Control, drought and drought pretreated with NAP are indicated by CTR, D and D + NAP, respectively. Data represent mean SD for three biological replicates. Significant differences P < 0.05 are indicated by different letters above the columns

To examine the role of *GmNFYA5* in regulation of tolerance to drought stress, 9 stress-related genes including 6 ABA-dependent genes (*ABI2*, *ABI3*, *NCED3*, *LEA3*, *RD29A* and *P5CS1*) and 3 ABA-independent genes (*DREB1A*, *DREB2A* and *DREB2B*) were assayed in three *35S:GmNFYA5* Arabidopsis lines in comparison with WT plants under drought, drought + NAP and control treatments. Under control conditions transcripts of *DREB1A*, *DREB2A*, *DREB2B*, *ABI2*, *ABI3*, *LEA3*, *RD29A* and *P5CS1* in *35S:GmNFYA5* plants were significantly higher than in WT plants; levels of *NCED3* transcripts were not different (Fig. 6). Moreover, all genes clearly produced much higher transcript levels in *35S:GmNFYA5* plants than in the WT under drought stress (Fig. 7). However, NAP significantly suppressed the drought-induced transcripts of all genes, especially transcripts of *ABI2*, *ABI3*, *LEA3*, *RD29A* and *P5CS1* in the drought + NAP treatment (Fig. 7E-I).

### Tolerance of transgenic soybean plants to drought stress

**Fig. 8:**
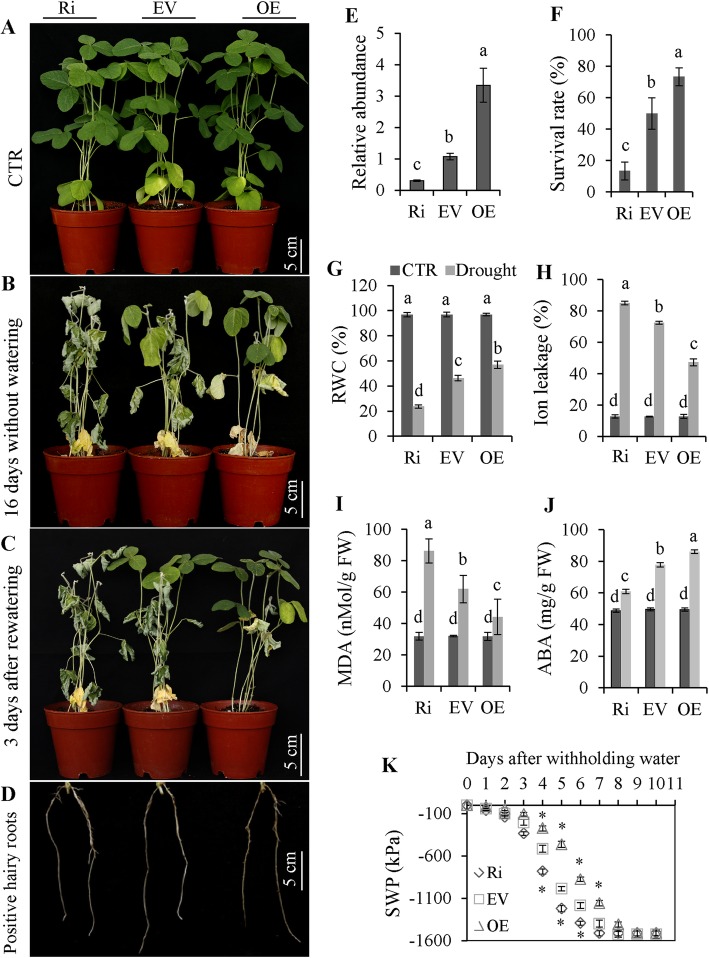
Drought tolerance in transgenic soybean plants. (A-D) Drought tolerance was evaluated in transgenic soybean plants. Transgenic soybean plants with positive hairy roots were transferred to plastic pots containing a mixture of peat and vermiculite (1:1, v/v) and grown for 7 days. Water deprivation for 16 days was followed by re-watering for 3 days. The hairy roots phenotype is shown in Fig. 8D. Drought resistance of transgenic soybean plants was assayed by the capability to resume growth when returned to normal conditions after drought stress. Scale bar, 5 cm. (E) Relative transcript of *GmNFYA5* was detected in three transgenic soybean lines. Transcript of *GmNFYA5* in EV plants was set at 1.0, and the expression levels were normalized to that of *GmCYP2*. (F-K) Survival rate, RWC, ion leakage, MDA, ABA concentration and SWP in transgenic soybean lines. Data represent mean SD for three biological replicates (n = 18). Significant differences P < 0.05 are indicated by different letters above the columns

Three transgenic soybean lines OE, EV and RNAi were generated by *A. rhizogenes*-mediated hairy roots transformation (Fig. 8D) to investigate the functions of *GmNFYA5* in soybean. Relative mRNA levels of *GmNFYA5* in OE plants measured by qRT-PCR were significantly higher than in plants with the EV, which in turn were higher than in RNAi plants (Fig. 8E). Then the plants that had positive hairy roots were planted into the soil for 7 days to establish the normal growth. These plants were used to explore the drought tolerance following deprivation of water.

The assay showed that the rate of decline in SWP was fastest in RNAi lines with minimum levels in RNAi, EV and OE lines being reached at 7, 8 and 9 days after initiation of drought treatment (Fig. 8K). Assuming that ABA induced in the roots was transferred to other tissue [33], the ABA concentration in soybean leaves was measured after withholding water for 7 days. The ABA concentration of OE plants was higher than in EV plants, and the opposite effect was detected in RNAi plants under drought stress. There was no difference among the lines under control conditions (Fig. 8J).

RNAi plants displayed severe wilting by withholding water for 16 days, with less extreme wilting in EV plants and a near-healthy appearance of OE plants (Fig. 8b). Under drought stress, there were much higher levels of ion leakage and MDA and decreased RWC in RNAi plants relative to EV plants, and opposite effects for OE plants (Fig. 8G-I). After re-watering for 3 days, survival of OE plants was significantly higher than that of EV plants, whereas nearly all RNAi plants were unable to recover (Fig. 8C and Fig. 8F).

### Transcription profiles of stress-related genes in transgenic soybean lines

**Fig. 9:**
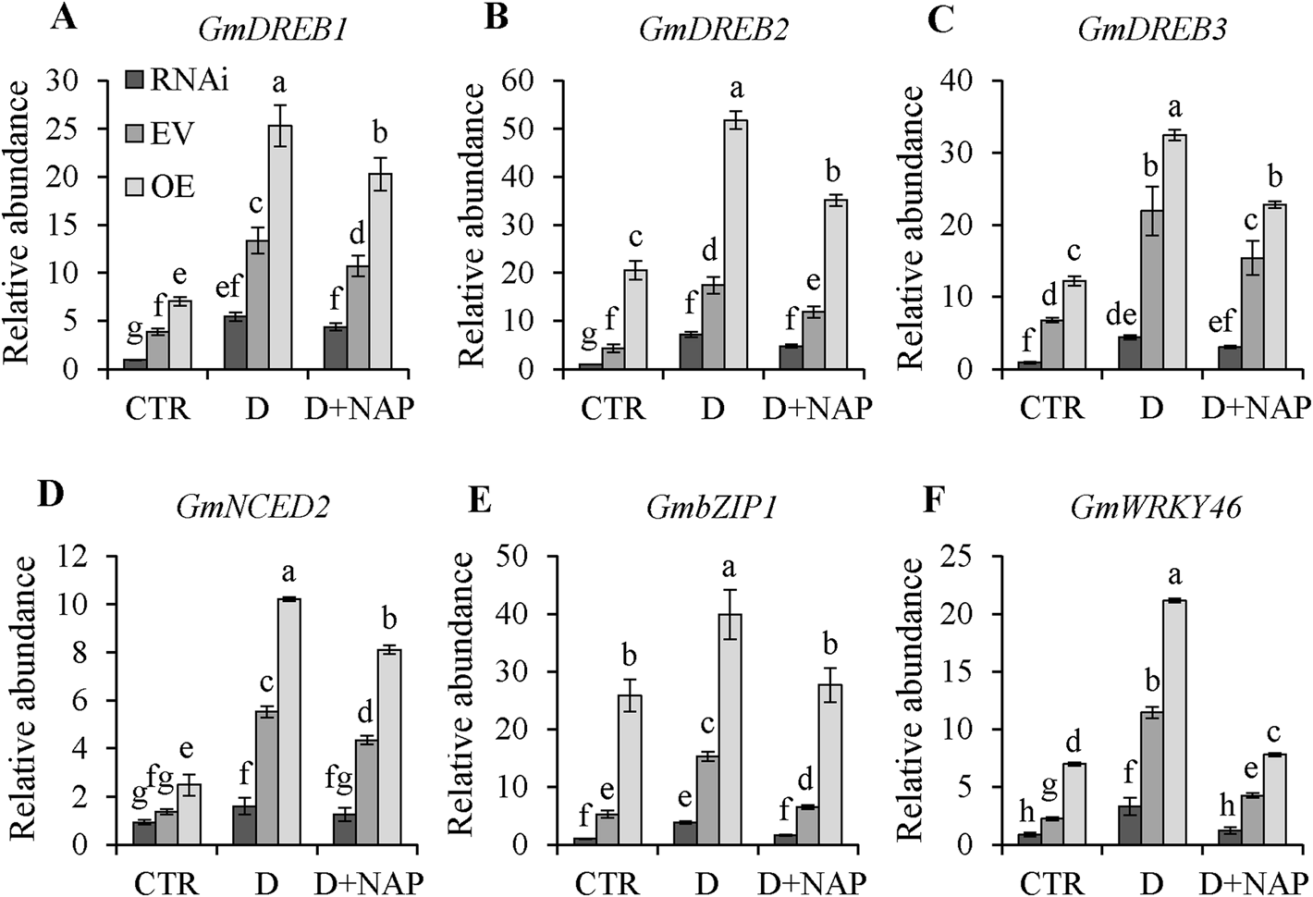
Relative transcript levels of *GmDREB1*, *GmDREB2*, *GmDREB3*, *GmNCED2*, *GmWRKY46* and *GmbZIP1* in transgenic soybean and EV plants under three conditions. Relative transcript level is indicated by the *vertical* column and normalized to that of *GmCYP2*. Transcript level of stress-related genes in EV plants under normal condition was set at 1.0. Control, drought and drought + NAP are indicated by CTR, D and D + NAP, respectively. Data represent mean SD for three biological replicates. Significant differences P < 0.05 are indicated by different letters above the columns

The transcript levels of 6 stress-responsive genes including 3 ABA-dependent genes (*GmDREB1*, *GmDREB2* and *GmDREB3*) and 3 ABA-independent genes (*GmWRKY46*, *GmNCED2* and *GmbZIP1*) were analyzed by qRT-PCR with treatments of control, drought and drought + NAP. Under control conditions, significantly higher expression levels of *GmDREB1*, *GmDREB2*, *GmDREB3*, *GmWRKY46* and *GmbZIP1* were detected in soybean OE hairy roots, and opposite effects were obtained for RNAi lines compared with EV lines. *GmNCED2* transcript showed no significant difference between RNAi and EV plants (Fig. 9A-F). Drought-induced expression levels of all 6 genes were significantly higher in OE lines, but much lower in RNAi lines compared with those in EV lines. NAP suppressed the levels of drought-induced transcripts of all genes, especially *GmWRKY46* and *GmbZIP1* (Fig. 9E and F).

### Transcriptional activation assays in Arabidopsis protoplasts

**Fig. 10:**
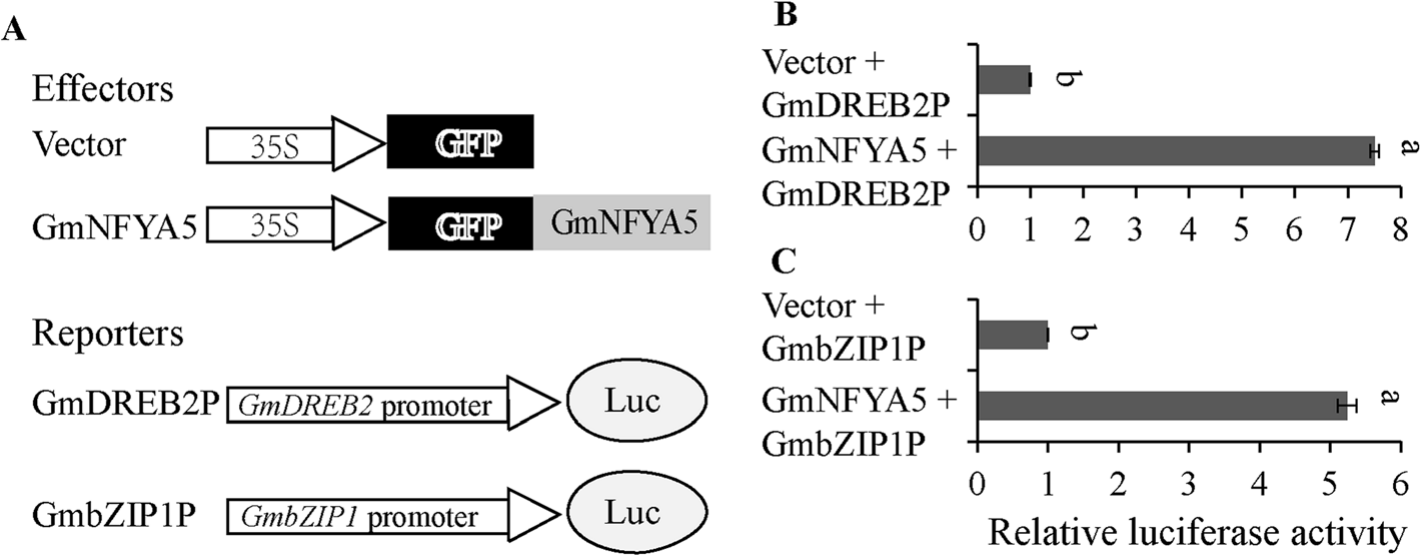
GmNFYA5 increased LUC reporter gene activity by binding the promoters of *GmDREB2* and *GmbZIP1*. (A) The structures of effector and reporters. (B) and (C) Ratio of LUC to REN indicates the activity of GmNFYA5 on the transcript of the *GmDREB2* and *GmbZIP1* promoters. Data represent mean SD for three biological replicates. Significant differences P < 0.05 are indicated by different letters above the columns

The promoters of 15 stress-responsive genes were analyzed by the PLACE program (http://www.dna.affrc.go.jp/PLACE/signalscan.html). One to six CCAAT elements were found in each gene (Table S2). Two stress-related genes (*GmDREB2* and *GmbZIP1*) were selected to determine whether GmNFYA5 could bind to the promoters in vivo in an Arabidopsis protoplast transient expression system. Recombinant pGreenII 0800:*GmDREB2p/GmbZIP1p* vector was co-transformed into Arabidopsis protoplasts with an empty p16318GFP vector or a p16318GFP:*GmNFYA5* vector (Fig. 10A). Protoplasts expressing GFP-GmNFYA5 exhibited significantly higher expression levels of LUC reporter gene compared with the GFP (Fig. 10B and C).

## Discussion

Drought is the most widespread and damaging abiotic stress [3]. Multiple transcriptional cascades mediate gene regulation under drought stress [34, 35]. Transcription factor genes induced in these cascades in turn regulate related downstream genes to resist the effects of drought stress. Our results showed that most NF-YA members responded to drought stress in soybean (Fig. 1A), consistent with previous observations [36]. Among the NF-YA genes the transcript level of *GmNFYA5* was the highest following drought treatment. Tissue-specific expression analysis showed that transcript abundance of *GmNFYA5* in roots was higher than in other tissues except the leaves (Fig. 2A), which implied that *GmNFYA5* was connected with drought tolerance. Likewise, several genes conferring drought tolerance in transgenic plants maintained their highest transcript abundance in roots under normal conditions [11, 27, 37].

In addition to being induced by drought stress, *GmNFYA5* transcript levels were up-regulated by ABA and suppressed by NAP (Fig. 1B and C), indicating that *GmNFYA5* transcription in response to drought depended on ABA signaling cross-talk. Under drought stress, the levels of ABA rise and suppress the activity of phosphatase 2Cs, followed by stomatal closure via phosphorylation and other pathways [38–40]. As expected, overexpression of *GmNFYA5* conferred drought tolerance to transgenic Arabidopsis plants via the ABA-dependent pathway (Fig. 5). Under drought treatment, overexpression of *GmNFYA5* increased the expression level of *NCED3* to enhance ABA biosynthesis (Fig. 5D and Fig. 7D), and increased ABA accumulation led to reduced stomatal apertures (Fig. 5H H and Fig. 5I), causing reduced water loss from leaves (Fig. 5F). Consistent with these results, the SWP of *35S:GmNFYA5* lines declined more slowly than in the WT (Fig. 5G). Reports that overexpression of *GmNFYA3* [31], *Cdt-NFYC1* [11], *RING-H2* [41] and *GmTGA17* [42] conferred increased ABA sensitivity and drought tolerance in transgenic lines, were confirmed in this study (Fig. 3). Moreover, some physiological indices, especially the SWP, were not reported in previous reports [6, 27, 32, 37].

As an important signaling intermediate, ABA controls the expression of many stress-induced genes [43, 44], such as *ABI2*, *ABI3*, *LEA3*, *RD29A* and *P5CS1*, which functioned as positive regulators of drought tolerance [45–48]. These genes maintained higher transcript levels in *35S:GmNFYA5* Arabidopsis plants compared with WT plants under normal and drought conditions (Fig. 7E-I). With the introduction of NAP into the drought treatment, reduced transcripts levels of these genes proved that ABA played a key role in regulating drought tolerance of plants. However, *GmNFYA5* overexpression functioned in enhancing drought tolerance via other pathways by up-regulating *DREB1A*, *DREB2A* and *DREB2B* (Fig. 7A-C), overexpression of which improved drought tolerance of Arabidopsis through an ABA-independent pathway [49–52]. Most importantly, we showed that overexpression of *GmNFYA5* led to significantly higher seed yield of *35S:GmNFYA5* Arabidopsis plants compared with WT plants under drought conditions (Fig. 6).

*GmNFYA5* functioned in positive regulation of drought tolerance in transgenic soybean plants; RNAi in those plants caused significant changes in several physiological indices (Fig. 8). The functions of *GmNFYA3* and *GmNFYB1* were previously studied only in transgenic Arabidopsis seedlings [32, 37]. In OE soybean plants, ABA-dependent genes (*GmbZIP1* and *GmWRKY46*) and ABA-independent marker genes (*GmDREB1*, *GmDREB2* and *GmDREB3*), which functioned in up-regulating drought tolerance in plants [45, 49, 51, 53, 54], maintained higher transcription levels compared with EV lines, whereas opposite effects occurred in RNAi plants (Fig. 9). The results showed that *GmNFYA5* conferred drought tolerance in transgenic Arabidopsis and soybean plants via both ABA-dependent and ABA-independent pathways.

One to six CCAAT *cis*-acting elements were detected in the promoters of all the marker genes used in this study (Table S2). Transcriptional activation assays showed that the promoters of *GmDREB2* and *GmbZIP1* were bound by GmNFYA5 to enhance the expression level of the LUC reporter gene in vivo (Fig. 10 b and c). The results give further insights into the regulation of drought tolerance by *GmNFYA5* through both ABA-dependent and ABA-independent pathways in Arabidopsis and soybean.

## Conclusions

Transgenic soybean and Arabidopsis plants overexpressing *GmNFYA5* exhibited enhanced drought tolerance as determined by physiological indices and transcript levels of drought-related genes. *GmNFYA5* should be a positive gene that can increase drought resistance and therefore has potential for use in molecular breeding of soybean.

## Methods

All experiments were performed in three biological replications.

### Isolation of *GmNFYA5*

Total RNA was isolated from soybean (*Glycine max* L. Merr.) cultivar Williams 82 by a previously described method [37]. The full-length cDNA of *GmNFYA5* was obtained by PCR with KOD-Plus DNA polymerase (TOYOBO, Japan) and the following primers: F 5′-GTAAGTGCGACTCTAAGCAAGCCT-3′ and R 5′-TAATGTAAATGAGCCAAGGATGACT-3′. The amplified products for sequencing were purified and cloned into the pEASY-Blunt vector (TransGen, China).

### Plant growth and treatments

Cultivar Williams 82 was grown in plastic pots (15 cm diameter, 20 cm depth) containing a mixture of peat and vermiculite (1:1, v/v) in a greenhouse at 28/18 °C day/night, 70% relative humidity and a 14/10 h light/darkness photoperiod [55]. Twenty-day-old seedlings were used for evaluation of expression patterns. For drought treatment, the whole plant was removed, washed and placed in a laminar flow hood for gradual drought exposure for 12 h [11]. For ABA treatment, the roots were subjected to 100 μM ABA for 12 h. For both treatments, the soybean leaves were collected at 0, 1, 2, 4, 8 and 12 h timepoints and immediately frozen by liquid nitrogen for isolation of RNA. To understand whether ABA was involved in drought-induced transcription of *GmNFYA5*, detached leaves were placed in H_2_O for 1 h to eliminate the influence of the wound stress, and then treated with distilled water or 1 mM naproxen (ABA synthesis inhibitor) for 3 h, followed by drought treatment for 2 h [11]. Leaves floated in H_2_O for the entire period constituted the normal control. The soybean leaves were collected and immediately frozen by liquid nitrogen for isolation of RNA.

To assay transcript levels of *GmNFYA5* in various tissues, the roots, stems, cotyledon, leaves and apical buds of 20-day-old soybean seedlings were sampled and immediately frozen by liquid nitrogen for isolation of RNA. Roots, stems, cotyledon, leaves, apical buds, flower buds and flowers of 50-day-old soybean plants were also sampled and immediately frozen for isolation of RNA. All the experiments were carried out in a greenhouse at 28/18 °C day/night, 70% relative humidity and a 14/10 h light/darkness photoperiod.

### Generation and drought treatment of *35S:GmNFYA5* Arabidopsis lines

The CDS of *GmNFYA5* was cloned into the *Nco*I site of a vector named pCAMBIA1302 and driven by CaMV35S promoter using primer set 5′-GGGACTCTTGACCATGATGAAGAACTTATGTGAG-3′ and 5′-TCAGATCTACCATGGCCATAAGGACTGATAGACG-3′. The pCAMBIA1302:*GmNFYA5* construct was introduced into *Agrobacterium tumefaciens* strain GV3101 which was used to infect Arabidopsis using the floral dip method. Positive transgenic Arabidopsis lines were screened by Hygromycin (Roche, Germany) to select T_1_ plants and T_2_ homozygotes.

Transgenic Arabidopsis lines (*35S:GmNFYA5–1*, *35S:GmNFYA5–2* and *35S:GmNFYA5–5*) and ecotype Columbia-0 (WT) seedling were used in this study. Seeds were surface-sterilized with 70% ethanol and thrice washed with sterile water, followed sterilization with 1% sodium hypochlorite for 15 min and again washing three times with sterile water. The seeds were sown on half-strength Murashige and Skoog medium (1/2 MS, 2% sucrose, 0.8% agar). After 2 days at 4 °C in darkness they were placed in a tissue culture room at 22 °C and 70% relative humidity with a 16/8 h light/darkness photoperiod. For drought treatment, 3-week-old seedlings which had been transferred to plastic pots (8 cm in length, width and depth) containing a mixture of peat and vermiculite (1:1, v/v) for 7 days were deprived of water until they became wilted, after which they were irrigated and recovered for 7 days.

To investigate the transcript levels of marker genes under conditions of control, drought and drought + NAP in *35S:GmNFYA5* Arabidopsis plants, leaves of 3-week-old *35S:GmNFYA5* and WT seedlings were placed into H_2_O for 1 h to eliminate the influence of the wound stress, following by placement in H_2_O or 1 mM NAP solution for 3 h, and then transferred to a laminar flow hood for 2 h as drought treatment. The leaves of *35S:GmNFYA5* and WT seedlings floated in H_2_O for the entire period were the normal control. Leaves were sampled and immediately frozen by liquid nitrogen for isolation of RNA. All the treatments were performed in a greenhouse at 22 °C and 70% relative humidity with a 16/8 h light/darkness photoperiod.

To further explore the functions of *GmNFYA5*, *35S:GmNFYA5* Arabidopsis lines were investigated under normal and drought conditions. For the normal control, 3-week-old *35S:GmNFYA5* and WT Arabidopsis plants were planted with well-watered treatment and 0.35 g H_2_O g^− 1^ dry soil (soil water potential is − 70 kPa) was maintained as the soil water content. For drought treatment, Soil water content was deprived of water to 0.20 g H_2_O g^− 1^ dry soil (soil water potential is − 280 kPa). The pots in both treatments were weighed daily and adjusted with water to maintain the target soil water potential until harvest [56]. All the treatments were performed in a greenhouse at 22 °C and 70% relative humidity with a 16/8 h light/darkness photoperiod.

The soybean seeds were provided by Dr. Li-Juan Qiu, Institute of Crop Science, Chinese Academy of Agricultural Sciences. The seeds of Arabidopsis were purchased from ABRC (https://abrc.osu.edu/researchers).

### Subcellular localization

The coding sequence (CDS) of *GmNFYA5* without the termination codon was fused in frame to the N-terminus of GFP in the vector p16318GFP, and ligated with *Bam*HI site to generate a p16318GFP:*GmNFYA5* fusion construct under the control of CaMV35S promoter using primer set 5′-TATCTCTAGAGGATCCATGAAGAACTTATGTGAG-3′ and 5′-TGCTCACCATGGATCCCATAAGGACTGATAGACG-3′. The CDS of *GmNFYA3* encoding a nuclear-localized protein [31] was cloned into the *Eco*RI site of a vector pLVX-IRES-mCherry using primer set 5′-TCTATTTCCGGTGAATTCATGCAAACTGTTTATCTT-3′ and 5′-ACTAGTCTCGAGGAATTCAACTTTAAGGTTGCAGCA-3′. The GmNFYA3:mCherry fusion protein was used as a nuclear marker. Arabidopsis protoplasts were prepared as described [57]. Transfected protoplasts were incubated in darkness at 22 °C for 16–18 h, GFP fluorescence signals were observed with a confocal laser scanning microscope (Zeiss, LSIM 700) [58].

### Promoter:GUS analysis

The promoter of *GmNFYA5* was amplified from the DNA of soybean cultivar Williams 82 with primers F 5′-AAGAGGAACACAGAAGTCTATGAGT-3′ and R 5′-GCACATCAGATTCAGAGGAAGTCCC-3′. The products were introduced into a reconstructive pCAMBIA1305 vector (GFP coding region was replaced by GUS coding region) incorporating *Eco*RI and *Nco*I sites with the forward primer 5′-CCATGATTACGAATTCAAGAGGAACACAGAAGTC-3′ and reverse primer 5′-CTCAGATCTACCATGGCTCACATAAGTTCTTCAT-3′. The construct was introduced into *A. tumefaciens* strain GV3101 and transferred into Arabidopsis Col-0 plants by the floral dip method. Positive transgenic Arabidopsis lines were screened by Hygromycin to obtain homozygous lines.

### Germination and root growth assays

Sterilized seeds were sown on 1/2 MS medium with 8–10% PEG 6000 (PEG) and 0.5–0.8 μM ABA respectively and placed in a tissue culture room at 22 °C and 70% relative humidity with a 16/8 h light/darkness photoperiod after stratification at 4 °C for 2 d in darkness. The germination rates were recorded every 12 h until completion. To investigate root growth of the *35S:GmNFYA5* Arabidopsis lines, 3-day-old seedlings were exposed to 1/2 MS medium with 10–12% PEG and 0.5–1 μM ABA. A week later, the root lengths were measured.

### *A. rhizogenes*-mediated transformation of soybean hairy roots

The CDS of *GmNFYA5* was inserted into the pCAMBIA3301 vector incorporating *Nco*I and *Bst*EII sites with the following primers: F 5′-GGACTCTTGACCATGATGAAGAACTTATGTGAG-3′ and R 5′-ATTCGAGCTGGTCACCCATAAGGACTGATAGACG-3′. A 635 bp synthetic RNAi hairpin fragment (Fig. S2) was introduced into the pCAMBIA3301 vector and ligated with the same restriction sites. The pCAMBIA3301:*GmNFYA5*, pCAMBIA3301 empty vector and pCAMBIA3301:RNAi*-GmNFYA5* construct were introduced into *A. rhizogenes* K599 and used to infect the hypocotyls of 5-day-old soybean seedlings in a tissue culture room at 28/18 °C day/night, 70% relative humidity and a 14/10 h light/darkness photoperiod, and then hairy roots were induced for 2 weeks [59]. The positive hairy roots transformants were screened using a QuickStix kit for PAT/bar (EnviroLogix, America) and qRT-PCR. Transgenic soybean lines having positive hairy roots were named OE-*GmNFYA5* (OE), empty vector (EV) and RNAi-*GmNFYA5* (RNAi) respectively and transferred to plastic pots (11 cm depth, 13.5 cm diameter) containing a peat and vermiculite mixture (1:1, v/v) to grow for 7 days, followed by deprivation of water until wilting.

To analyze the transcription of drought-related genes under conditions of drought and drought + NAP in transgenic soybean plants, three transgenic hairy roots lines were placed into H_2_O for 1 h to eliminate the wound stress, following by placement in H_2_O or 1 mM NAP solution for 3 h, prior to transfer to a laminar flow hood for 2 h as drought treatment. Roots floated in H_2_O for the entire period were the normal control. All treatments were carried out in a tissue culture room at 28/18 °C day/night, 70% relative humidity and a 14/10 h light/darkness photoperiod. Roots were sampled and immediately frozen by liquid nitrogen for isolation of RNA.

### Analysis of transcript levels

Transcript levels were measured by qRT-PCR performed with TransStart Top Green qPCR SuperMix (TransGen, China) (20 μl) according to the manufacturer’s instructions on an Applied Biosystems 7500 real-time PCR system. Gene-specific primers designed by https://biodb.swu.edu.cn/qprimerdb/ and used for qRT-PCR assays are listed in Table S1.

### Measurement of water loss

Leaves of 3-week-old Arabidopsis lines were detached, and the weight was measured every 30 min in a tissue culture room at 22 °C and 70% relative humidity. Percentages of initial fresh weight at 9 timepoints were used to represent water loss in transgenic and WT plants.

### ABA concentration and stomatal aperture analysis

Following deprivation of water for 7 day, the ABA concentrations of leaves of soybean and Arabidopsis plants were measured by means of an ABA ELISA assay kit (Jiancheng, China) as described [60].

Leaves of 3-week-old *35S:GmNFYA5* and WT Arabidopsis seedlings were treated for 3 h in stomatal opening buffer (5 mM MES, 10 mM KCl, 50 mM CaCl_2_, pH 5.6) as described previously [61]. The leaves were then transferred to H_2_O or 10 μM ABA solution for 2 h in a greenhouse at 22 °C and 70% relative humidity. Stomatal apertures of stomatal complexes with no surrounding mesophyll cells were measured.

### Measurement of malondialdehyde (MDA), relative water content (RWC) and ion leakage

RWC and ion leakage were measured in 3-week-old Arabidopsis plants following drought treatment for 14 days. MDA contents, RWC and ion leakage in transgenic soybean plants were measured following drought treatment for 16 days. MDA contents were measured and calculated as described previously [58, 62]. RWC and ion leakage were determined as described previously [63, 64].

### Measurement of soil water potential (SWP)

When the transgenic soybean and Arabidopsis plants were shut off water supply, the SWP was measured in drought treatment trials until it reduced to the minimum level using WP4-T dewpoint meter (Decagon Devices, USA) according to the manufacturer’s instructions. WP4-T dewpoint meter had been used in research due to its accuracy in several reports [56, 65].

### Transcriptional activation assays

The promoters of *GmDREB2* (ABA-related gene) and *GmbZIP1* (ABA-unrelated gene) were cloned into the LUC reporter vector pGreen II 0800 containing the Renilla luciferase (REN) gene driven by the CaMV 35S promoter and used as an internal control. The effector and reporter plasmids were extracted and introduced into Arabidopsis protoplasts using PEG4000-mediated transformation. The assays were performed as described [6].

### Statistical analysis

All the measurements in this study were replicated three times biologically. Variance analyses of all data were preformed using SPSS Statistics 22 (IBM, USA) for a completely randomized design model. Duncan’s tests was used to evaluate differences among plant lines or treatments at *P* = 0.05.

## Supplementary information

**Additional file 1: Fig. S1.** Sequence alignment of the conserved domains of GmNFYA5 and members of NF-YA family in Arabidopsis. (A) Sequence alignment of the conserved domains in GmNFYA5 and 10 members of NF-YA family in Arabidopsis. Two subdomains and the linker are underlined. Asterisks indicate critical amino acids. (B) Phylogenetic analysis of *GmNFYA5* with 10 members of NF-YA family in Arabidopsis. The unrooted neighbor joining tree was constructed using MEGA 7.0.

**Additional file 2: Fig. S2.** Sequence of RNAi-*GmNFYA5*. The hairpin structure is composed of three sequences: the positive sequence of RNAi-*GmNFYA5* in blue, the reverse complementary sequence in green, and intron of *GmNFYA5* in purple. Restriction sites *Nco*I and *Bst*EII are in red above the horizontal line. The sequence was inserted into the pCAMBIA3301 vector to generate a pCAMBIA3301:RNAi*-GmNFYA5* construct.

**Additional file 3: Fig. S3.** Ion leakage in *35S:GmNFYA5* Arabidopsis plants at the seedling stage under normal and drought conditions. Data represent mean SD for three biological replicates. Significant differences at *P* < 0.05 are indicated by different letters above the columns.

**Additional file 4: Table S1.** List of primers for qRT-PCR used in this study.

**Additional file 5: Table S2.** Promoter sequence analysis of genes up-regulated by *GmNFYA5* in transgenic Arabidopsis and soybean lines.

## Data Availability

Soybean genes sequences in this research were downloaded from the plant genomics resource (https://phytozome.jgi.doe.gov/pz/portal.html) and National Center for Biotechnology Information (NCBI) (https://www.ncbi.nlm.nih.gov/). Arabidopsis genes sequences in this research were downloaded from The Arabidopsis Information Resource (TAIR) (http://www.arabidopsis.org/). The primers for qRT-PCR used in this research were designed in https://biodb.swu.edu.cn/qprimerdb/. The gene accessions and special primers for qRT-PCR are listed in Table S1. Arabidopsis plants and soybean plants used in this research were treated following the guidelines of ABRC and Dr. Li-Juan Qiu, respectively.

## Abbreviations

ABA: abscisic acid
GFP: Green fluorescent protein
MDA: malondialdehyde
NAP: naproxen
RWC: relative water content
