# Supplementary figures and images for: Overexpression of GmNFYA5 confers drought tolerance to transgenic Arabidopsis and soybean plants
Source: BMC Plant Biol. 2020 Mar 20;20:123. doi: 10.1186/s12870-020-02337-z (PMC7082914; doi:10.1186/s12870-020-02337-z)

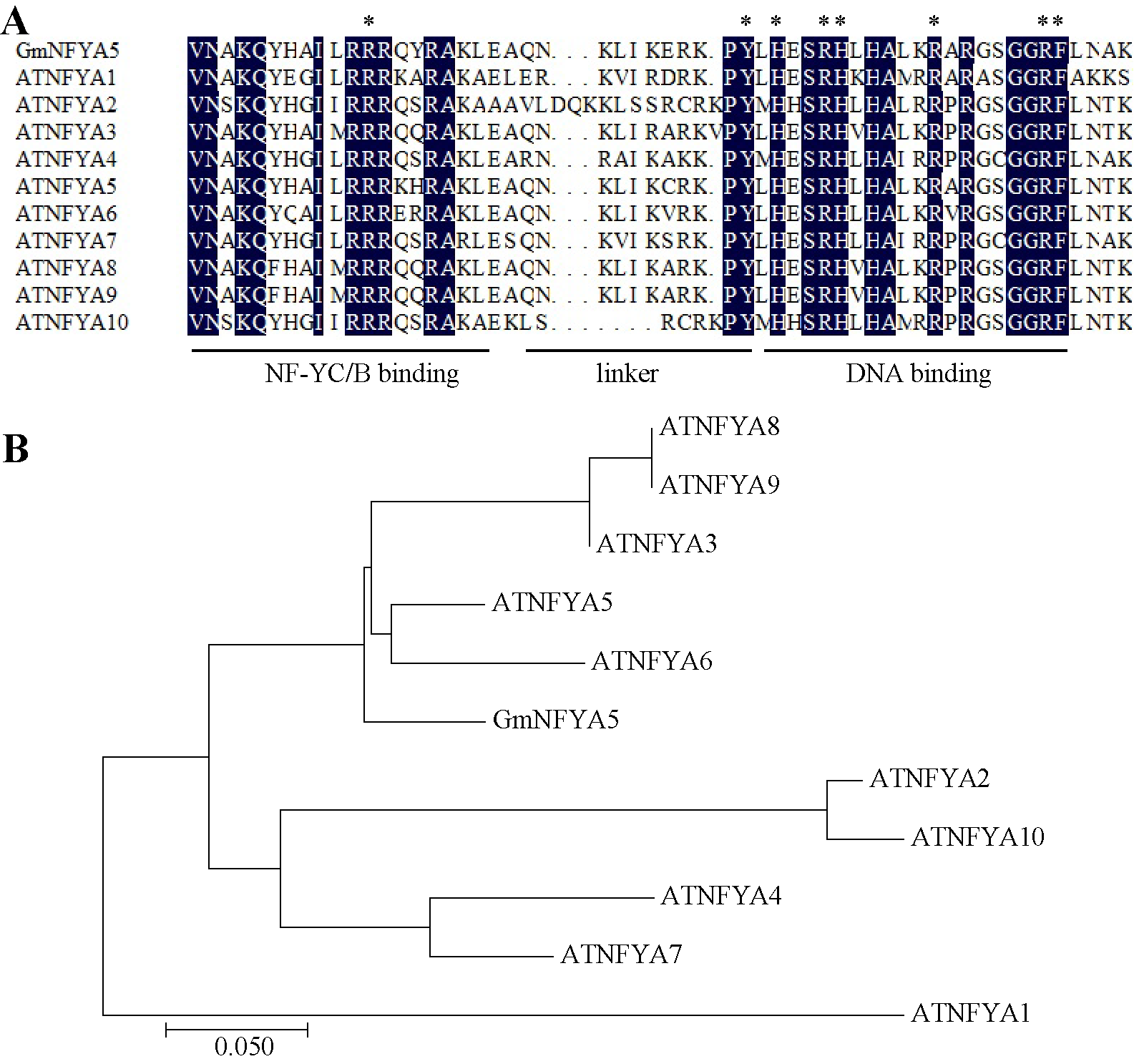

Supplement: Supplementary file 1 — Additional file 1: Fig. S1. Sequence alignment of the conserved domains of GmNFYA5 and members of NF-YA family in Arabidopsis. (A) Sequence alignment of the conserved domains in GmNFYA5 and 10 members of NF-YA family in Arabidopsis. Two subdomains and the linker are underlined. Asterisks indicate critical amino acids. (B) Phylogenetic analysis of GmNFYA5 with 10 members of NF-YA family in Arabidopsis. The unrooted neighbor joining tree was constructed using MEGA 7.0. [file 12870_2020_2337_MOESM1_ESM.tif]

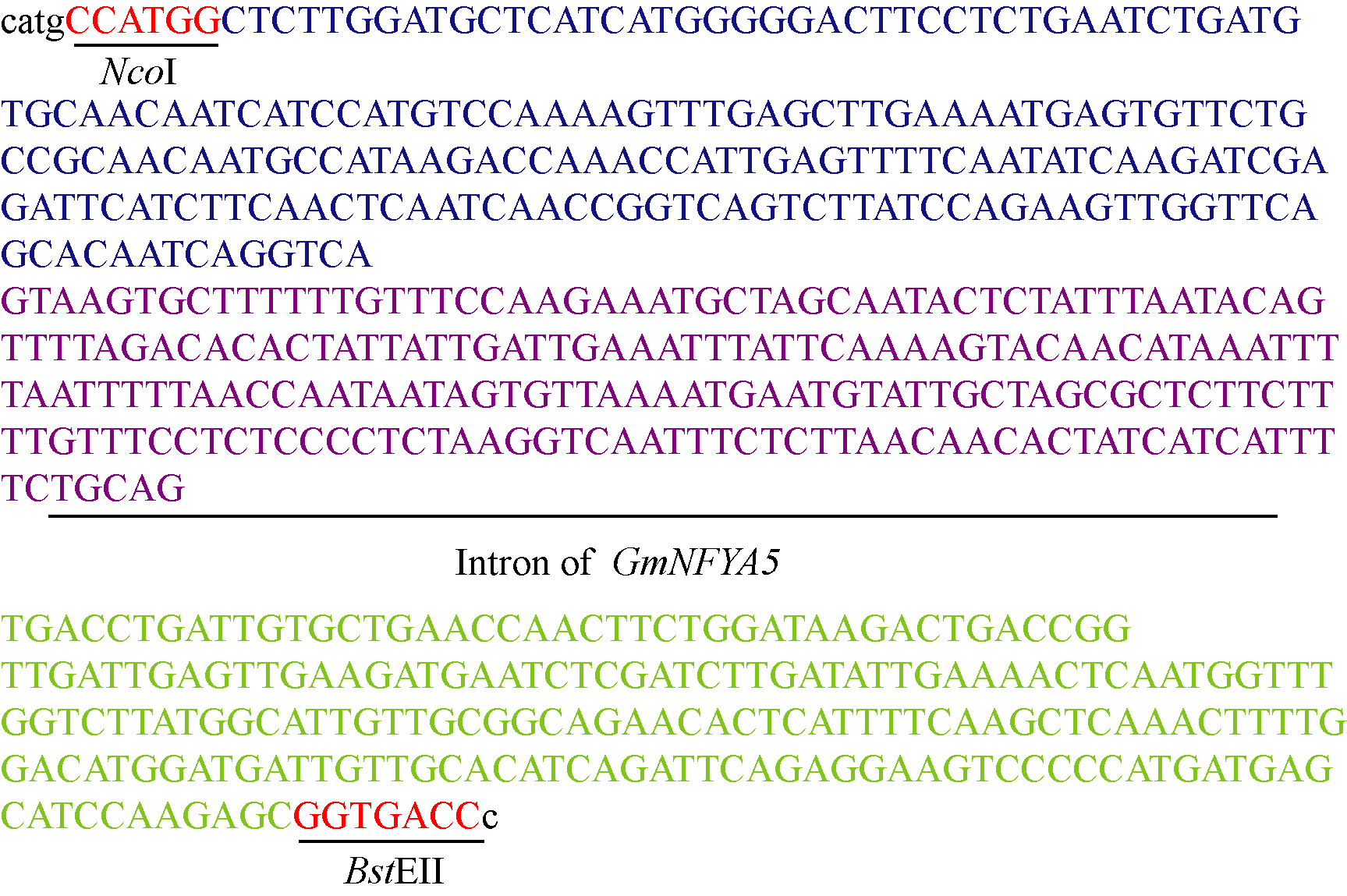

Supplement: Supplementary file 2 — Additional file 2: Fig. S2. Sequence of RNAi-GmNFYA5. The hairpin structure is composed of three sequences: the positive sequence of RNAi-GmNFYA5 in blue, the reverse complementary sequence in green, and intron of GmNFYA5 in purple. Restriction sites NcoI and BstEII are in red above the horizontal line. The sequence was inserted into the pCAMBIA3301 vector to generate a pCAMBIA3301:RNAi-GmNFYA5 construct. [file 12870_2020_2337_MOESM2_ESM.tif]

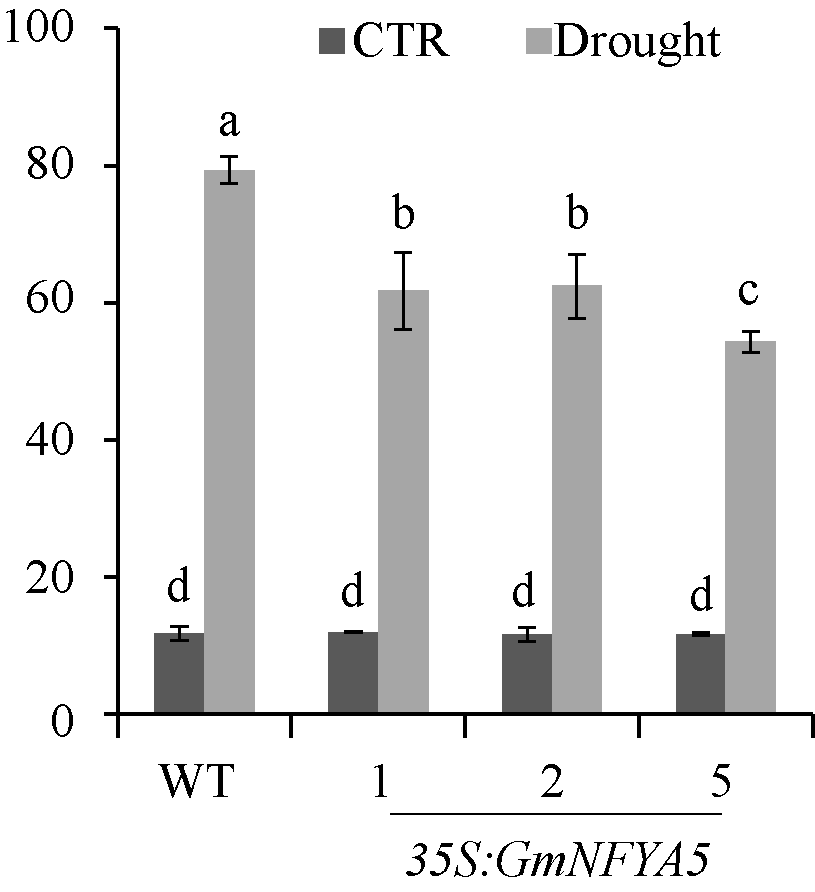

Supplement: Supplementary file 3 — Additional file 3: Fig. S3. Ion leakage in 35S:GmNFYA5 Arabidopsis plants at the seedling stage under normal and drought conditions. Data represent mean SD for three biological replicates. Significant differences at P < 0.05 are indicated by different letters above the columns. [file 12870_2020_2337_MOESM3_ESM.tif]
